# Sexing Bones: Improving Transparency of Sex Reporting to Address Bias Within Preclinical Studies

**DOI:** 10.1002/jbmr.4729

**Published:** 2022-11-13

**Authors:** Aikta Sharma, Lysanne V Michels, Andrew A Pitsillides, Julie Greeves, Lillian I Plotkin, Valentina Cardo, Natalie A Sims, Claire E Clarkin

**Affiliations:** ^1^ School of Biological Sciences University of Southampton Southampton UK; ^2^ Department of Comparative Biomedical Sciences Royal Veterinary College London UK; ^3^ Army Health and Performance Research, Ministry of Defence Andover UK; ^4^ Department of Anatomy, Cell Biology and Physiology School of Medicine, Indiana University Indianapolis IN USA; ^5^ Winchester School of Art University of Southampton Winchester UK; ^6^ Department of Medicine at St. Vincent's Hospital St. Vincent's Institute of Medical Research and The University of Melbourne Fitzroy Australia

**Keywords:** GENETIC ANIMAL MODELS, PRECLINICAL STUDIES, OSTEOPOROSIS, SEX STEROIDS, AGING

## Abstract

Despite knowledge that sexually dimorphic mechanisms regulate bone homeostasis, sex often remains unreported and unconsidered in preclinical experimental design. Failure to report sex could lead to inappropriate generalizations of research findings and less effective translation into clinical practice. Preclinical sex bias (preferential selection of one sex) is present across other fields, including neuroscience and immunology, but remains uninvestigated in skeletal research. For context, we first summarized key literature describing sexually dimorphic bone phenotypes in mice. We then investigated sex reporting practices in skeletal research, specifically how customary it is for murine sex to be included in journal article titles or abstracts and then determined whether any bias in sex reporting exists. Because sex hormones are important regulators of bone health (gonadectomy procedures, ie, ovariectomy [OVX] and orchidectomy [ORX], are common yet typically not reported with sex), we incorporated reporting of OVX and ORX terms, representing female and male mice, respectively, into our investigations around sex bias. Between 1999 and 2020, inclusion of sex in titles or abstracts was low in murine skeletal studies (2.6%–4.06%). Reporting of OVX and ORX terms was low (1.44%–2.64%) and reporting of OVX and ORX with sex uncommon (0.4%–0.3%). When studies were combined to include both sexes and OVX (representing female) and ORX terms (representing male), a bias toward reporting of female mice was evident. However, when the terms OVX and ORX were removed, a bias toward the use of male mice was identified. Thus, studies focusing on sex hormones are biased toward female reporting with all other studies biased in reporting of male mice. We now call upon journal editors to introduce consistent guidance for transparent and accessible reporting of murine sex in skeletal research to better monitor preclinical sex bias, to diversify development of treatments for bone health, and to enable global skeletal health equity. © 2022 The Authors. *Journal of Bone and Mineral Research* published by Wiley Periodicals LLC on behalf of American Society for Bone and Mineral Research (ASBMR).

## Introduction

It is well understood that the post‐pubertal skeleton exhibits sexual dimorphism and becomes anatomically and physiologically distinct in mammalian species, including humans. In women, sex differences are amplified with age and in the onset of degenerative skeletal pathologies such as osteoporosis.^(^ ^1^, ^2^ ^)^ Preclinical mouse models have proved to be extremely useful in improving our understanding of how sex steroids regulate sexual dimorphism, in identifying the genetic and molecular contributors of bone loss and in the development of new therapeutics.^(^ ^3^, ^4^, ^5^, ^6^ ^)^ The use of mouse models in studies of human skeletal physiology and pathology at the genetic, molecular, cellular, and tissue level have become increasingly common over the last several decades because of their short gestation period, high reproductive capacity, and their relatively low cost.^(^ ^7^ ^)^ Further, given the high genomic similarities between mice and humans, over other rodent models, the use of mice models in skeletal research also provides an opportunity to study the sexually dimorphic mechanisms that underpin bone cell function in skeletal growth and development in addition to the contributions of hormone and non‐hormonal factors involved in aging and pathology through gene manipulation approaches. However, aside from studies into the control of bone homeostasis by sex hormones, sex often remains unreported when it is not the primary focus of the study. To promote the inclusion of sex as a biological variable in research, the Sex and Gender Equity in Research (SAGER) guidelines, developed by an international panel in consultation with scientists and journal editors,^(^ ^8^ ^)^ were published with the aim to provide a comprehensive standardized procedure for reporting sex and gender information in study design, data analysis, results, and interpretations of findings. The guidelines provide researchers and authors with a tool to standardize sex and gender reporting in scientific publications, whenever appropriate. A failure to focus upon or address sex in preclinical research could lead to inappropriate generalizations of research findings and the unmonitored development of sex bias, which may ultimately result in the ineffective translation of findings into clinical practice.^(^ ^9^, ^10^ ^)^

## Sexual Dimorphism and Sex Bias

Preceding puberty, sex differences have been reported in clinical measurements of bone mass of the axial and appendicular skeleton after adjustments for age, nutrition, and physical activity.^(^ ^11^ ^)^ Boys have been reported to develop a larger periosteal perimeter than girls from mid‐puberty onward.^(^ ^12^, ^13^ ^)^ In contrast, girls experience less periosteal expansion but more endocortical apposition compared with boys. As a result, men form wider and stronger bones, with cortical bone generated farther from the neutral axis thereby providing increased resistance to bending. Consistent with this dimorphism, women exposed to arduous military training and female athletes exhibit a higher stress fracture prevalence than men, an incidence of ~9.2% and ~ 3% in military populations and ~9.7% and ~6.5% in athletes for women and men, respectively.^(^ ^14^ ^)^ There are also sexual dimorphic consequences on the skeleton as individuals age. Osteoporosis is more prevalent in postmenopausal women than men of the same age,^(^ ^1^ ^)^ and this has been the focus of much research in the field. However, with increasing life expectancy, age‐related osteoporosis is a growing problem for men.^(^ ^15^ ^)^

The development of skeletal sexual dimorphism is comparable between murine models and humans, with sex differences in bone mass established during puberty and associated with similar patterns of sex steroids produced.^(^ ^16^ ^)^ Male and female mice, like humans, also exhibit divergent bone traits, with male skeletons having greater cortical thickness, trabecular volume, trabeculae number, along with greater cortical and trabecular bone mass than females.^(^ ^17^, ^18^, ^19^, ^20^, ^21^ ^)^ Although the bones of female mice are overall smaller than males, female mice undergo earlier and greater appositional growth and present with a thicker periosteum, which has been recently linked to sex‐specific distinctions in gene expression profiles of cortical and intramedullary osteogenic cell populations.^(^ ^19^ ^)^ Female mice have also been shown to have higher osteoblast and osteoclast numbers on trabecular bone surfaces.^(^ ^22^ ^)^

Growth hormone (GH) and insulin‐like growth factor (IGF)‐1 have been shown to be critical regulatory determinants of the sex differences in bone mass in pubertal mice^(^ ^23^ ^)^ with IGF‐1 levels higher in male mice versus female mice during early puberty.^(^ ^24^ ^)^ Ovariectomy (OVX) in rodents induces both cortical and trabecular bone loss,^(^ ^22^, ^25^, ^26^, ^27^ ^)^ consistent with the impact of estrogen withdrawal in women.^(^ ^28^ ^)^ Rodent studies have also confirmed the importance of androgens in regulating the male skeleton, with orchidectomy (ORX) decreasing longitudinal and cortical growth.^(^ ^29^, ^30^, ^31^, ^32^ ^)^ Male mice lacking 3‐oxo‐5‐α‐steroid 4‐dehydrogenase 1, required for the conversion of testosterone to dihydrotestosterone, exhibit reduced cortical thickness, whereas female mice present with elevated cortical bone mass associated with elevated circulating androgen levels.^(^ ^33^ ^)^ Similarly, male mice deficient of the androgen receptor (AR) present with reductions in cortical and trabecular bone mass, through upregulated expression of receptor activator of NF‐κB ligand (RANKL) in male AR‐deficient osteoblasts.^(^ ^34^ ^)^ Our understanding of the role of sex hormones in regulating bone homeostasis has been deepened by the generation of mouse models that are deficient of the estrogen receptor (ER) and AR. ER‐α, for example, has been shown to be the key mediator of the protective function of estradiol in trabecular bone of both male and female mice.^(^ ^22^ ^)^ Bone adaptation in response to mechanical strain has been shown to require ER‐α alpha signaling in females, with ER‐α knockout osteoblasts unresponsive to strain in vitro compared with wild types.^(^ ^35^ ^)^ Further, the role of ER‐α in response to mechanical loading in vivo has been described as sexually dimorphic within both trabecular and cortical bone compartments.^(^ ^36^ ^)^

Sexual dimorphism of the skeleton is not restricted to sex‐steroid responses. Skeletal phenotypes of genetically altered mice, with deletion of factors not known to have a sex‐specific role in the skeleton, have also been noted, including in mice with lineage‐specific deletions. In many cases, a phenotype is only observed in females (eg, *OsxCre.Efnb2* ^*f/f*^ mice,^(^ ^37^ ^)^ *Dmp1Cre.Efnb2* ^*f/f*^ mice,^(^ ^38^ ^)^ *LysMCre.Krox20* ^*f/f*^ mice^(^ ^39^ ^)^), sometimes only in males (eg, *OcnCre.Vegf* ^*f/f*^ mice^(^ ^40^, ^41^ ^)^), and sometimes the phenotype differs profoundly between male and female mice (eg, *Dmp1Cre.Socs3* ^*f/f*^ mice,^(^ ^42^ ^)^ *Dmp1Cre.miR21* ^*f/f*^ mice,^(43^ ^)^ *TRAPCre.Panx1* ^*f/f*^ mice^(^ ^43^ ^)^). Skeletal sexual dimorphisms have also been documented in the femoral trabeculae of the wild‐type inbred mouse strains; C57BL/6, FVB, C3H/HeJ, and BALB/c with age‐related decline in cancellous bone volume occur more rapidly in female mice than male littermates.^(^ ^44^ ^)^ Although sex differences in bone formation were not observed, additional in vitro studies revealed that such sex‐specific loss in bone volume with age in wild‐type strains occurred because of the diminished osteogenic differentiation capacity of female bone marrow stromal cells. Sexual dimorphic mechanisms have also been reported in osteocytic control of TGF‐beta receptor II signaling, shown to influence the skeleton of male but not female mice under homeostatic conditions.^(^ ^45^ ^)^ The conditional deletion of vascular endothelial growth factor (VEGF) in osteocalcin‐expressing cells induces sexually dimorphic phenotypes with male mice exhibiting severe, prepubertal, cortical porosity linked to deficient mineralization driven by sex differences in matrix composition and sclerostin expression.^(^ ^40^ ^)^ Further, VEGF deletion in male and female long bone–derived osteoblasts results in sex‐specific extracellular matrix and genetic signatures.^(^ ^41^ ^)^ Sex differences have been reported to underlie osteoclast function^(^ ^46^ ^)^ and sexual dimorphism in fracture repair evidenced in wild‐type animals.^(^ ^47^ ^)^

Recently the prevalence of sexual divergence in phenotypic traits, including DXA‐derived indices of bone mass, length, and area, has been described in high‐throughput phenotype data from 2186 single‐gene knockout mouse lines generated as part of the International Mouse Phenotyping Consortium. These analyses confirmed a large proportion of traits in wild‐type and mutant mice were influenced by sex, thus illustrating the need to consider sex as a biological variable in murine skeletal studies.^(^ ^48^ ^)^ After this initial screening study, 220 select mouse lines were further phenotyped as part of the Knockout Mouse Project, with additional dimorphism in bone shape and cellular content identified by micro‐computed tomography, histomorphometry, and histological analyses (https://bonebase.lab.uconn.edu/).^(^ ^49^ ^)^ Despite these sex differences, a prevailing male sex bias in reporting remains in a wide range of pathological mouse models associated with preclinical studies of diabetes and cardiovascular disease while female bias predominates in studies of infectious diseases and cancer.^(^ ^50^ ^)^ While the prevalence of any sex bias existing within preclinical bone research remains unclear, what is apparent and of concern is that these studies in particular typically either do not report their findings in both sexes, do not report the sex at all, or have combined sexes in their analysis.

Given the growing relevance of sexually dimorphic mechanisms that underpin bone homeostasis and disease onset, along with the historic reporting bias in mouse sex across multiple fields, we have sought to assess patterns in the reporting of murine sex in skeletal research articles. This Perspective describes a systematic PubMed search examining skeletal research articles that include murine sex in either the article's title or abstract, published between 1999 and 2020 and has also evaluated whether any bias in sex reporting exists. Because sex is typically not reported in conjunction with gonadectomy procedures, we have also assessed the reporting of OVX and ORX (independently of sex) in murine skeletal articles and utilized OVX and ORX data to represent female and male mice, respectively, to inform investigations into bias. Finally, we compared sex bias in murine skeletal studies that do not involve gonadectomy.

## Murine Sex Reporting in Journal Title or Abstract From 1999–2009 and 2010–2020

A systematic search of the PubMed database examined inclusion of sex in the title or abstract of scientific articles published using mice between January 1999 and December 2020. A total of 6,909,612 articles were included in period 1 (published between January 1999 and December 2009), and 11,961,640 articles were included in period 2 (published between January 2010 and December 2020). After excluding articles that did not report original research (see Fig. 1 for exclusion criteria), data mining was performed on the remaining 4,160,407 (period 1) and 8,028,460 articles (period 2), respectively.

**Fig. 1:**
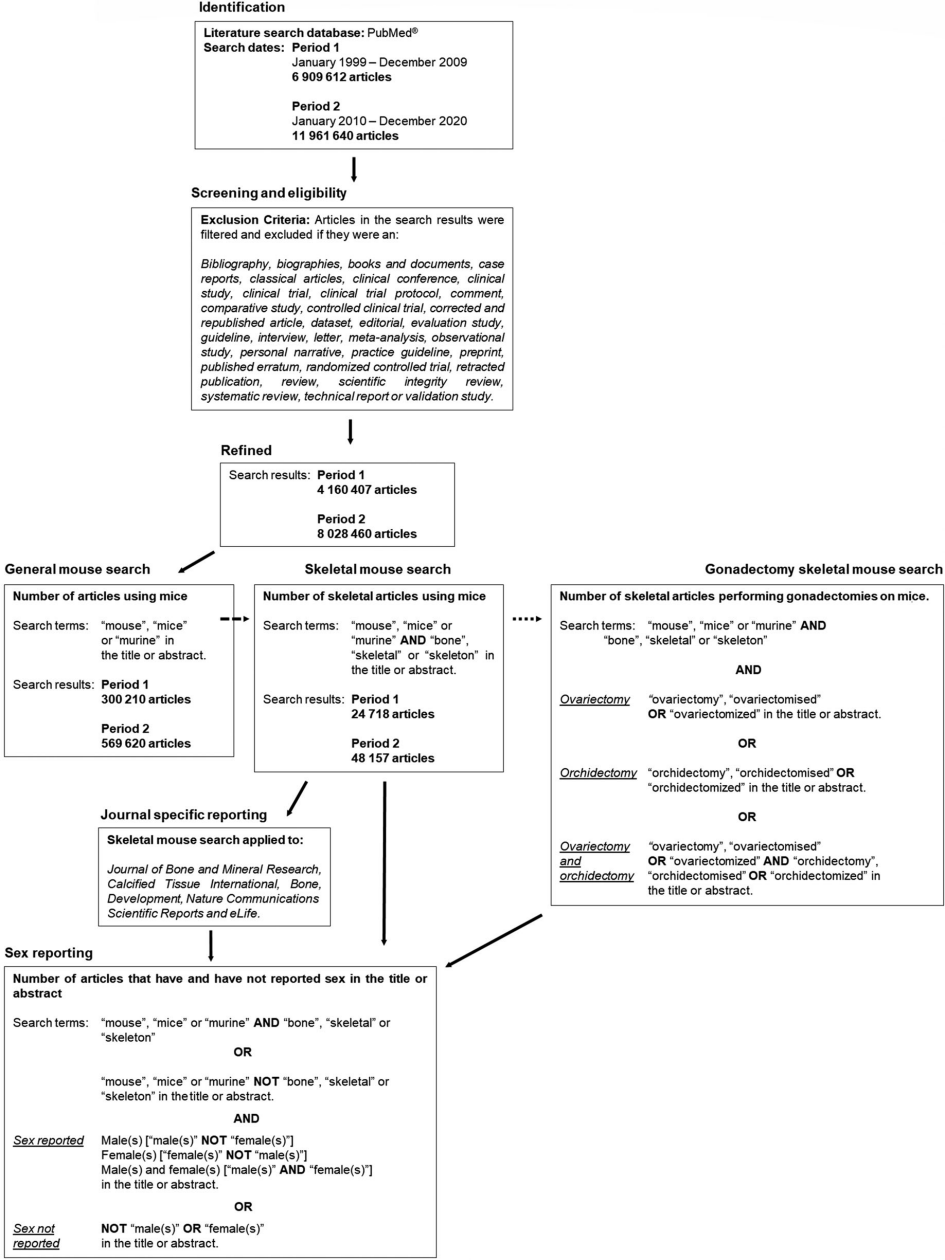
Flow diagram detailing the systematic method for reviewing the reporting and non‐reporting of sex in the title or abstract of murine skeletal articles using PubMed.

For period 1, a total of 300,210 articles included the terms “mouse,” “mice,” or “murine” in the titles or abstracts (termed “general mouse search”) with 24,718 of these also including the terms “bone,” “skeleton,” or “skeletal” (termed “skeletal mouse search”) (Fig. 2*A*). In period 1, of the 24,718 articles discovered by the “skeletal mouse search,” the sex of mice (wherein “male(s)” and/or “female(s)” were mentioned) was reported in the titles or abstracts of 646 articles (2.61%).This mining approach was also applied to identify the number of articles that utilized mice but did not include the terms “skeleton,” “skeletal,” or “bone” in their title or abstract (Fig. 2*A*, termed “non‐skeletal research”) for the same period. Of the 275,492 articles identified in the “general mouse search,” 6256 (2.27%) reported sex. This is similar to the low reporting identified in skeletal mouse studies.

**Fig. 2:**
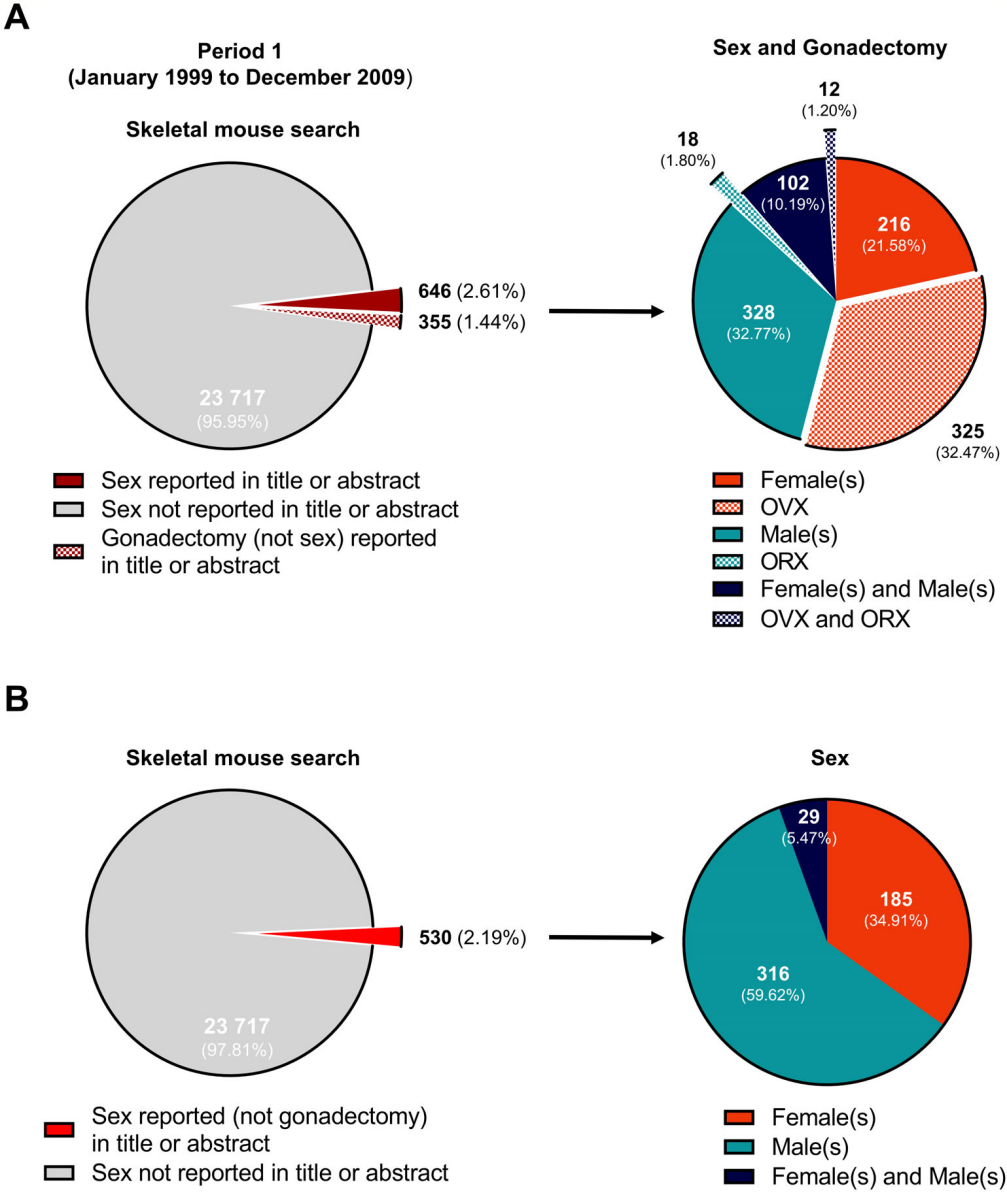
Historical reporting and non‐reporting of sex and gonadectomy procedures in the title or abstract of murine skeletal articles published in period 1. A total of 24,718 scientific articles, published between January 1999 and December 2009 (period 1), were obtained in the “skeletal mouse search” using PubMed (*A*, left). Articles were screened for the inclusion of sex (male(s), female(s) or male(s) and female(s)) and the reporting of gonadectomy procedures (ovariectomy, OVX, orchidectomy, or ovariectomy and orchidectomy, OVX and ORX), independently of sex, in the corresponding titles or abstracts. Refined screening of articles in which sex or specified gonadectomy procedures were disclosed in the titles or abstracts were then used for the evaluation of sex bias and bias in the prevalence of gonadectomy procedures (*A*, right). Articles reporting gonadectomy in the title or abstract were excluded from the “skeletal mouse search” to resolve the extent of general sex reporting in murine skeletal studies (*B*, left) ahead of sex bias assessment (*B*, right). Data are expressed as pie charts with the number and percentage of articles in each segment displayed.

We predicted that sex would not be used in conjunction with the terms OVX and ORX, and accordingly undertook additional searches to determine the reporting of OVX/ORX in the article's title or abstract either independently or in combination with sex. In the “skeletal mouse search,” where sex was reported, only 116 articles (0.46%) included both sex and gonadectomy (comprising OVX, ORX, or both). As we predicted, a more common approach was to use OVX and ORX without sex; 355 articles (1.44%) were identified in these searches (Fig. 2*A*).

Given that OVX/ORX are typically used and reported without sex, we included these articles to represent female and male mice for our analyses (Fig. 2*A*). Overall, among the studies that used gonadectomy, we discovered a bias toward the use of female mice; 541 papers (54.05%) reported female and/or OVX; 346 papers (34.57%) reported male and/or ORX, and 114 (11.39%) articles reported both male and female with OVX and ORX together.

To investigate whether any bias in reporting of male and female mice is evident in non‐gonadectomy studies, articles including the terms OVX and ORX in the titles or abstracts were removed from the analyses (Fig. 2*B*). Of the 530 articles identified as “non‐gonadectomy skeletal studies,” a bias toward reporting male mice was evident; 316 articles reported the use of males (59.62%), 185 reported the use of females (34.91%), and 29 (5.47%) reported the use of both sexes.

To examine if the reporting of sex and the presence of sex bias has changed in the last 20 years, we also analyzed the literature published within period 2 (Fig. 3*A*). The number of articles identified in the “skeletal mouse search” increased to 48,157 articles in period 2, with 1954 articles (4.06%) reporting sex in the titles or abstracts, an increase in sex reporting from period 1. In the “non‐skeletal research” for the same period, of the 521,463 articles identified, 16,637 (3.19%) reported sex in the titles or abstracts. Where sex was reported, only 173 articles (0.35%) included sex and gonadectomy together. The use of OVX and ORX without sex was higher than in period 1 and evident in 1318 articles (2.73%).

**Fig. 3:**
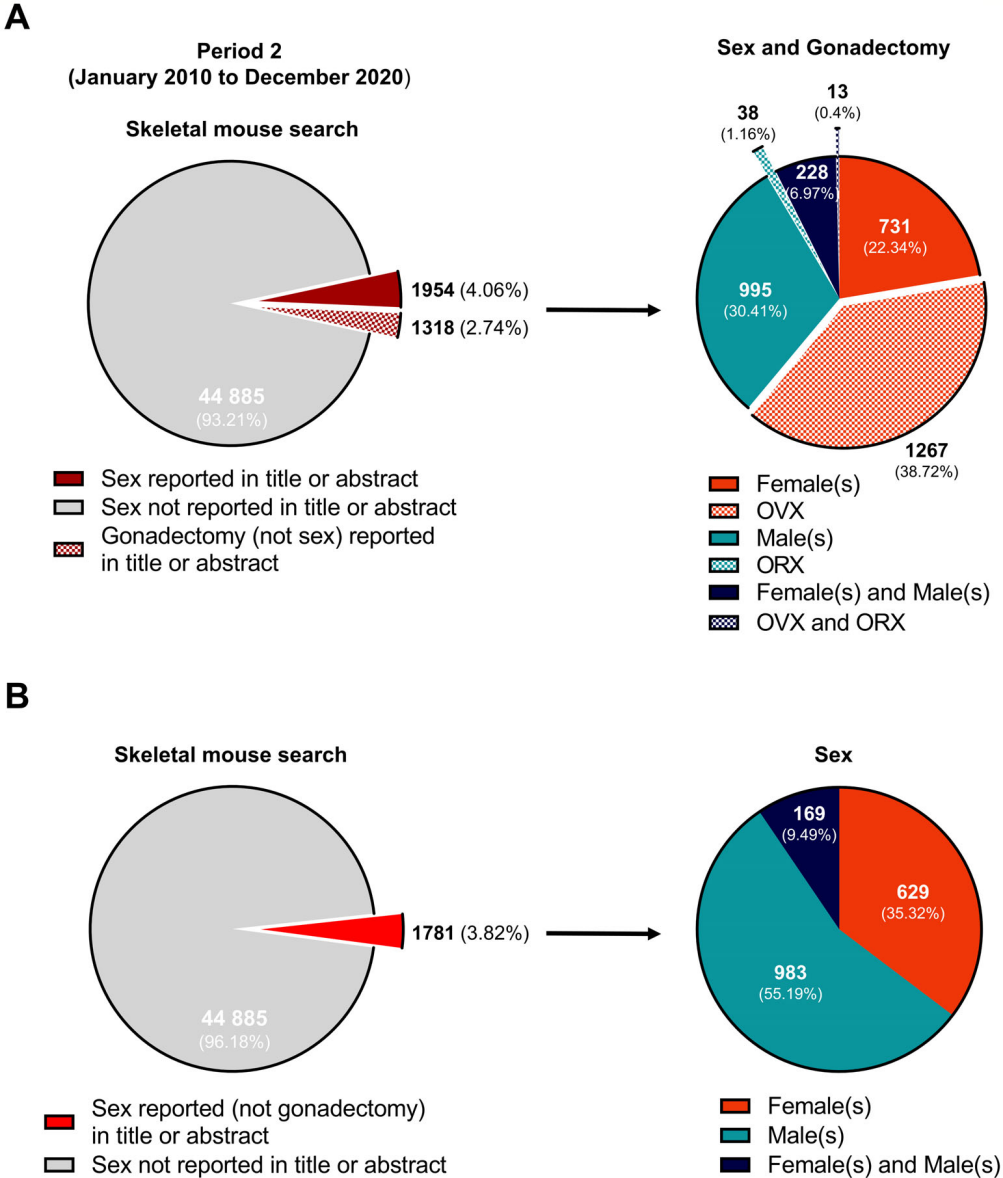
Reporting and non‐reporting of sex and gonadectomy procedures in the title or abstract of murine skeletal articles published in period 2. A total of 48,157 scientific articles, published between January 2010 and December 2020 (period 2), were obtained in the “skeletal mouse search” using PubMed (*A*, left). Search results were subsequently filtered for the disclosure of sex of the mice and the reporting of gonadectomy procedures, without sex, in the titles or abstracts (*A*, left). Identified articles were subjected to analysis of sex bias and bias in gonadectomy procedure prevalence (*A*, right). Generalized reporting and non‐reporting of sex in the titles or abstracts of articles was examined after the exclusion of articles reporting gonadectomy procedures from search results (*B*, left) before sex bias evaluation (*B*, right). Data are expressed as pie charts with the number and percentage of articles in each segment displayed.

In comparison to period 1, in skeletal research studies with gonadectomy reported, we also found an increased bias toward reporting the use of female mice in period 2: 1998 articles (61.08%) reported female and/or OVX in the titles or abstracts, whereas 1033 articles (30.41%) reported male and/or ORX, and 241 (7.37%) articles reported male and female together with OVX and ORX. When the terms OVX and ORX reported in the title of abstract were removed from analyses (Fig. 3*B*), there was a slight decrease in the male bias compared with period 1. Reporting of male mice remained higher in period 2, being present in 983 papers (55.19%), 629 papers (35.32%) for female mice, and 169 articles (9.48%) including both sexes.

## Sex Reporting—A Comparison Between Bone‐Specialist and General Science Journals

We next assessed sex reporting in specialist (*JBMR, Calcified Tissue International,* and *Osteoporosis International*) and non‐specialist (*Development, Nature Communications, Scientific Reports,* and *eLife*) journals publishing original scientific articles on murine skeletal studies (Table 1).

**Table 1:** Reporting of Sex in the Title or Abstract of Articles Identified in the “Skeletal Mouse Search” Published in Selected Journals During Period 1 and Period 2

	Period 1 January 1999–December 2009					Period 2 January 2010–December 2020			
	Total no. of articles	No. of articles in which sex was reported				Total no. of articles	No. of articles in which sex was reported		
*Journal of Bone and Mineral Research* (Wiley)	506	41 (8.1%)	Male(s)	18	*Journal of Bone and Mineral Research* (Wiley)	750	61 (8.1%)	Male(s)	26
			Female(s)	19				Female(s)	20
			Male(s) and Female(s)	4				Male(s) and Female(s)	15
*Bone* (Elsevier)	351	13 (3.7%)	Male(s)	0	Bone (Elsevier)	732	55 (7.5%)	Male(s)	24
			Female(s)	11				Female(s)	20
			Male(s) and Female(s)	2				Male(s) and Female(s)	11
*Calcified Tissue International* (Springer)	106	14 (13.2%)	Male(s)	6	*Calcified Tissue International* (Springer)	156	27 (17.3%)	Male(s)	10
			Female(s)	7				Female(s)	12
			Male(s) and Female(s)	1				Male(s) and Female(s)	5
*Osteoporosis International* (Springer)	7	0 (0%)	Male(s)	1	*Osteoporosis International* (Springer)	46	5 (10.9%)	Male(s)	2
			Female(s)	0				Female(s)	3
			Male(s) and Female(s)	0				Male(s) and Female(s)	0
*Development* (The Company of Biologists)	216	1 (0.4%)	Male(s)	1	*Development* (The Company of Biologists)	173	0 (0%)	Male(s)	0
			Female(s)	0				Female(s)	0
			Male(s) and Female(s)	0				Male(s) and Female(s)	0
					*Nature Communications* (Springer Nature)	306	5 (1.63%)	Male(s)	0
								Female(s)	4
								Male(s) and Female(s)	0
					*Scientific Reports* (Springer Nature)	1087	35 (3.22%)	Male(s)	26
								Female(s)	6
								Male(s) and Female(s)	3
					*eLife* (eLife Science Publications)	142	0 (0%)	Male(s)	0
								Female(s)	0
								Male(s) and Female(s)	0

In *JBMR* for period 1, 8.1% of “skeletal mouse search” articles (41 of 506) described the sex of the mice used in the titles or abstracts. This was similar for period 2 with sex being stated in 8.13% of articles. Improvements in the reporting of sex in the titles or abstracts of articles published in the journal *Bone* were evident over time, with 3.7% (13 of 351) increasing to 7.51% (55 of 732) between period 1 and period 2, respectively. Such trends were evident in *Calcified Tissue International*, which had a higher initial reporting than the aforementioned specialist journals, at 13.21% (14 of 106 articles) for period 1, increasing to 17.31% (27 of 156) for period 2. In *Osteoporosis International*, 0% of articles (0 of 7) identified in the “skeletal mouse search” reported sex in the titles or abstracts in period 1. In period 2, this had increased by 10.9% (5 of 42). Overall, this indicates an increase in sex reporting in bone‐specialist journals, although it is still a low level of reporting.

For the journal *Development*, in period 1, the reporting of sex following the “skeletal mouse search” was lower than the specialized skeletal journals, with 0.5% of identified articles including sex in the titles or abstracts (1 of 216). For period 2, this was 0% (0 of 173). As *Nature Communications, Scientific Reports*, and *eLife* were established after 2010, these data mining procedures were exclusively applied to those articles published within the timeframe of period 2. For *Nature Communications*, 1.95% of articles (6 of 307) mentioned sex, whereas only one specifically included both “male(s) and “female(s)” in the corresponding article title or abstract. *Scientific Reports* reported sex in the titles or abstracts of 0.03% of articles identified from the “skeletal mouse search” (35 of 1087). In the newest journal, *eLife*, 0% of articles (0 of 142) disclosed the sex of the mice in the abstract or title.

## Addressing Preclinical Sex Bias—Journals, Funders, and Beyond

Health inequalities linked to sex have existed for many years, but the clinical consequences of such inequity have been exposed during the ongoing COVID‐19 pandemic. As a result, it is equally important to establish whether sex bias is also present across our own research fields and experimental studies. Within the bone field, particularly concerning osteoporosis research, the study of postmenopausal women and female animal models is common and, as a consequence, it is evident that sex reporting is generally greater in number of skeletal‐focused journals, including *JBMR*, than non‐specialist journals. Despite this, more broadly, the lack of sex reporting in the titles or abstracts continues to be uncommon practice across all of the journals that we have analyzed.

Following our review, we have identified a sex bias in reporting the use of female mice specifically linked to OVX. This female bias is unsurprising and aligns with clinical and societal pressures linked to osteoporosis postmenopause. Further, historically, osteoporotic drugs have (i) been tested predominantly in female OVX animal models^(^ ^51^, ^52^ ^)^ and (ii) clinically in female postmenopausal cohorts.^(^ ^53^, ^54^, ^55^ ^)^ The pathogenesis of male osteoporosis continues to be poorly understood despite being a growing concern in our aging societies. Interestingly, we have reported that when gonadectomy studies are removed from our searches, the murine skeletal articles that remain report use of male mice more often than female. This finding suggests that either (i) male mice are preferentially selected for these experiments or (ii) that reporting of male mice in the title or abstract is deemed more relevant than reporting of females.

It is plausible that the sex of the mice may have been reported elsewhere in the article, for example, in the methodology section rather than the title or the abstract, resulting in the exclusion of some articles from our analyses. Although this is a limitation of our approach, it is important to note that the titles and abstracts of all research articles are the only sections that are readily accessible to all readers. In this regard, given that sex differences are a key finding, this information should be first presented in the title or abstract rather than being reported later in the article. Accordingly, we now recommend an urgent adjustment in sex‐reporting practices across our research community, with support from journals to allow for wider dissemination of important sex‐specific findings. Indeed, laboratory data now being generated from wild‐type, transgenic, and knockout animals, particularly where mechanisms underlying phenotypic sex differences are being compared within the same study, are proving extremely informative as previously described.

As part of an initiative to increase rigor and reproducibility in biomedical research, global funders including the NIH, EU Horizon, and the National Health and Medical Research Council (Australia) have instructed that sex is to be considered as a biological variable. More recently, NIH and EU Horizon have announced plans that “require grant applicants to describe how they will balance of male and female animals and cells in preclinical studies, unless sex‐specific inclusion is unwarranted.” According to the NIH, “both sex and gender play a role in how health and disease processes differ among individuals and consideration of these factors in research studies informs the development and testing of preventative and therapeutic interventions.”^(^ ^56^, ^57^ ^)^ As of January 25, 2016, the NIH expected that sex as a biological variable was be factored into research designs, analyses, and reporting in vertebrate animals and human studies, with the exception of studies in which the use of only one sex is strongly justified.

To assess the effectiveness of the policy change by NIH, we have used the NIH Research Portfolio Online Reporting Tools Expenditures and Results (RePORTER) database to search for funded applications in which terms related with sex/gender inclusion are listed in the title or abstract. The RePORTER is an electronic tool that allows users to search a repository of both intramural and extramural NIH‐funded research projects and access publications and patents resulting from NIH funding since 1985 (Table 2). Of the 2,608,050 proposals funded since 1985, 130,254 include the word “bone” in the title or abstract. The results of this search indicated that when proposals funded between 2016 and 2022 were compared with those funded between 1985 and 2015, there was a 2.5% increase in the number of proposals that included “bone” in the abstract or title, with a 1.7% increase in applications that, in addition to the term “bone,” also included “male” and “female,” suggesting a positive effect of the NIH rule. Similar increases (0.5%) were found when “female” and “woman” and “women” and by 0.3% when the terms “male” and “man” and “men” were included in addition to “bone.” The percentage of applications that included these terms, however, is still low and ranges from 0.6% to 1.1% of all bone‐related proposals. Although results of these searches do not reflect the content of the applications, (which we assume follow the NIH guidelines around sex/gender), these findings highlight how a change in the directives from funders can make investigators more aware of the significance of sex/gender comparisons in experiments, which is reflected by inclusion of sex article title and/or abstract.

**Table 2:** NIH‐Funded Applications Before and After the Establishment of the Requirement to Include Both Sex/Genders in All Studies

		Terms searched					
Years	Total no. of articles	“Male” and “female”	“Bone”	“Bone” and “male” and “female”	“Bone” and “female” and “woman” and “women”	“Bone” and “male” and “man” and “men”	“Bone” and “sex” or “gender”
1985–2022	2,608,050	39,399 (1.5%)	130,524 (5.0%)	2700 (2.07%)	959 (0.7%)	541 (0.41%)	832 (0.64%)
1985–2015	2,113,529	25,090 (1.2%)	95,861 (4.5%)	1631 (1.7%)	565 (0.6%)	321 (0.3%)	646 (0.7%)
2016–2022	494,521	14,309 (2.9%)	34,663 (7.0%)	1069 (3.1%)	394 (1.1%)	220 (0.6%)	186 (0.5%)

We would now encourage skeletal journals to align their guidelines around sex reporting to reflect the changes in NIH policies, for example, by providing clearer guidance to authors around sex reporting in preclinical studies. To help improve and standardize transparency of sex reporting, SAGER‐based guidance could, for example, be a mandatory requirement for publication with (i) the title and abstract of the article specifying the sex/gender of the research subjects, (ii) using the term “sex” and not “gender”” in animal studies, (iii) including the origin and sex of cells or tissues, (iv) routine reporting of data that is disaggregated by sex, and (v) the acquisition and reporting of data from both sexes regardless of the findings.

Given the availability of funding now for sex‐focused research, publishers should be primed to support these policies and modify publishing guidelines as necessary for sex‐focused observations to be appropriately disseminated. Better reporting practices from preclinical work could have substantial benefits, enabling us to most effectively identify gaps in knowledge linked to either sex, to generate the most relevant sex‐specific research hypotheses in our laboratories, and ultimately to diversify and optimize treatment options available in the clinic.

## Disclosures

The authors declare no competing interests.

## Author Contributions

**Aikta Sharma:** Conceptualization; formal analysis; investigation; methodology; project administration; resources; software; validation; visualization; writing – original draft; writing – review and editing. **Lysanne Veerle Michels:** Formal analysis; investigation; methodology; resources; software; validation. **Andrew Pitsillides:** Writing – original draft. **Julie P Greeves:** Writing – original draft. **Lilian Plotkin:** Formal analysis; investigation; methodology; resources; software; visualization; writing – original draft. **Valentina Cardo:** Methodology; resources; writing – original draft. **Natalie A Sims:** Methodology; visualization; writing – original draft. **Claire E Clarkin:** Conceptualization; funding acquisition; methodology; project administration; resources; supervision; validation; visualization; writing – original draft; writing – review and editing.

### Peer Review

The peer review history for this article is available at https://publons.com/publon/10.1002/jbmr.4729.

## Data Availability

Data derived from public domain resources
